# Supplementary material for: Mapping the energy level alignment at donor/acceptor interfaces in non-fullerene organic solar cells
Source: Nat Commun. 2022 Apr 19;13:2046. doi: 10.1038/s41467-022-29702-w (PMC9018783; doi:10.1038/s41467-022-29702-w)
Supplement: Supplementary file 1 — Supplementary Information [file 41467_2022_29702_MOESM1_ESM.pdf]

Supplementary Information

Mapping the energy level alignment at donor/acceptor interfaces in non-fullerene organic solar cells

Xian'e Li[1,*], Qilun Zhang[1], Jianwei Yu[2], Ye Xu[3], Rui Zhang[2], Chuanfei Wang[1], Huotian Zhang[2], Simone Fabiano[1], Xianjie Liu[1], Jianhui Hou[3], Feng Gao[2], Mats Fahlman[1,*]

[1]Laboratory of Organic Electronics, Department of Science and Technology (ITN), Linköping University, Norrköping SE-60174, Sweden.
[2]Biomolecular and Organic Electronics, Department of Physics, Chemistry and Biology (IFM), Linköping University, Linköping SE-58183, Sweden.
[3]Beijing National Laboratory for Molecular Sciences, State Key Laboratory of Polymer Physics and Chemistry, Institute of Chemistry, Chinese Academy of Sciences, Beijing 100190, China.
* Correspondence to: xiane.li@liu.se (X.L.); mats.fahlman@liu.se (M.F.)

Contents:

Supplementary Figures 1-17

Supplementary Tables 1-8

Supplementary Note 1

Supplementary References

Supplementary Figures 1-17

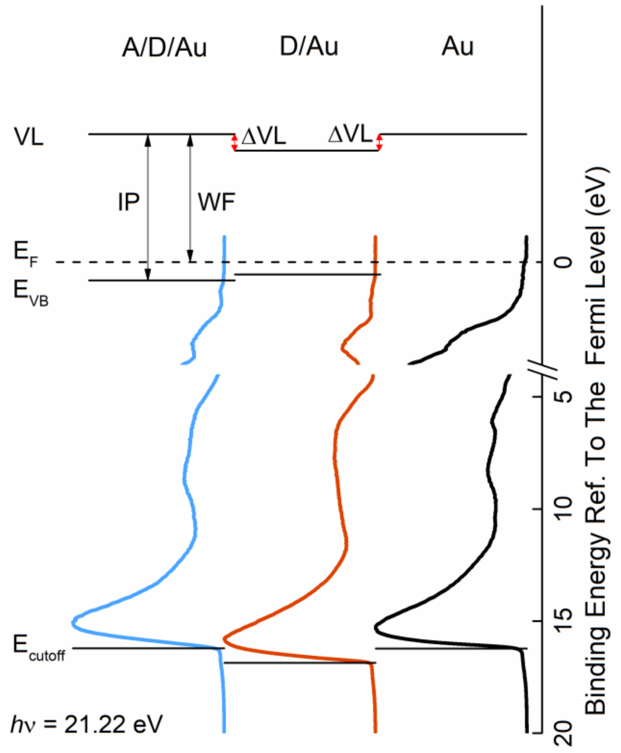

Supplementary Fig. 1 Schematic illustration for the determination of vacuum level shifts (ΔVL), or work function changes (ΔWF) at Au/Donor(D)/Acceptor(A) heterojunction interfaces by using ultraviolet photoelectron spectroscopy (UPS). UPS spectra are usually plotted as function of binding energy referenced to the Fermi level ($E_F = 0$ eV). The energy of secondary-electron cutoff (E_{cutoff}) is used to determine the VL or WF of each layer (WF = $h\nu - E_{cutoff}$). Ionization potential (IP) of each film is derived from the energy difference between the valence band (E_{VB}) edge and VL.

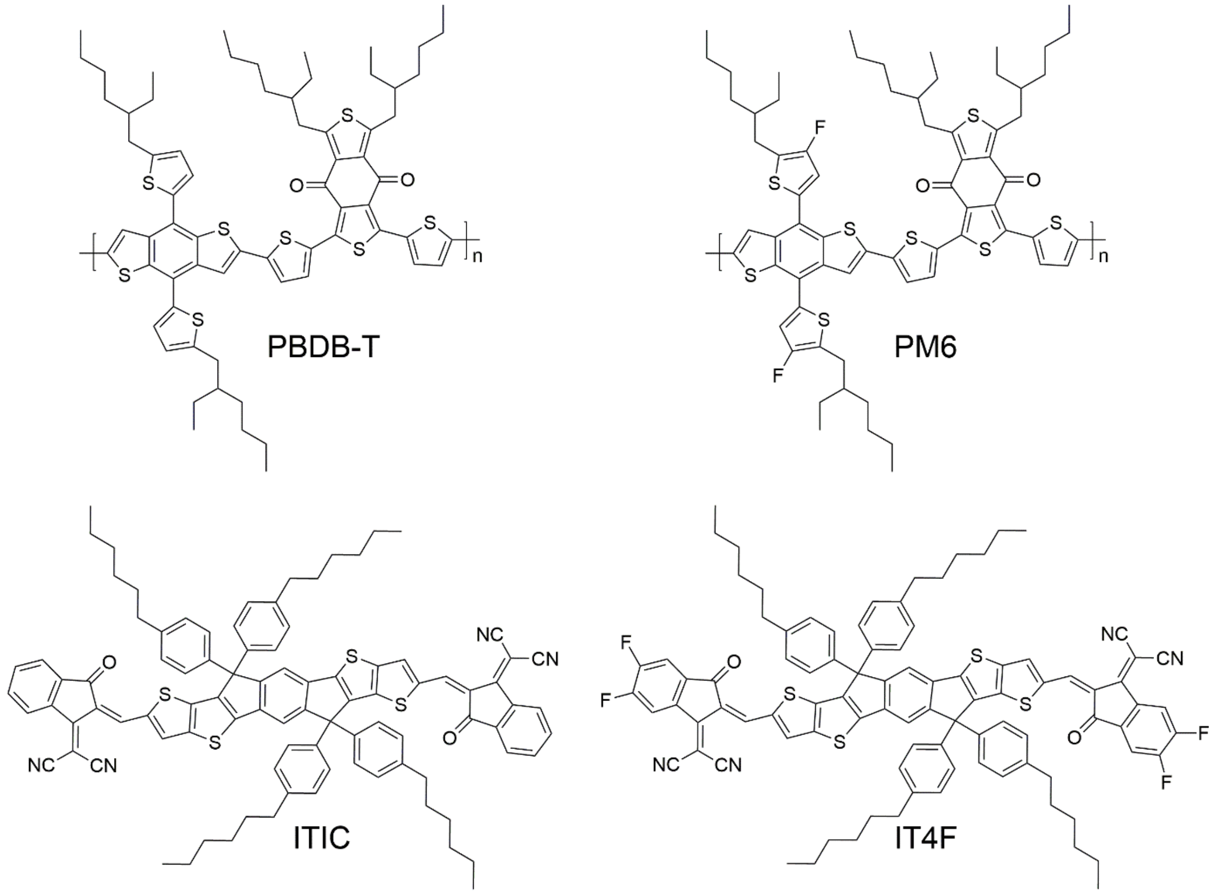

Supplementary Fig. 2 Molecular structures of four model materials: donors (PBDB-T, PM6) and acceptors (ITIC, IT4F).

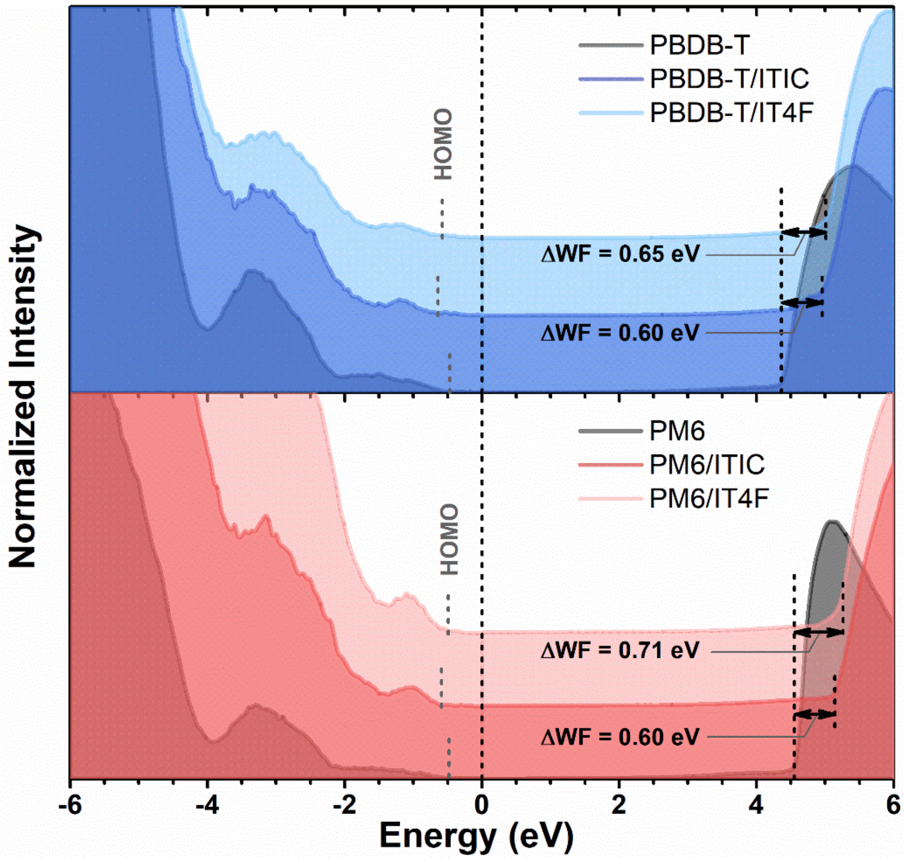

Supplementary Fig. 3 UPS spectra of neat donor films (PM6 or PBDB-T spin-coated on UVO-treated Au) and quasi-bilayer D-A films of ITIC or IT4F on PBDB-T or PM6 (PBDB-T/ITIC, PBDB-T/IT4F, PM6/ITIC, PM6/IT4F) in which the work function shifts between the neat donor layer and D-A bilayer (ΔWF) and their respective HOMO levels are labeled in the spectra. WFs of quasi-bilayer D-A films increase 0.60−0.71 eV compared to those of their respective neat donor films.

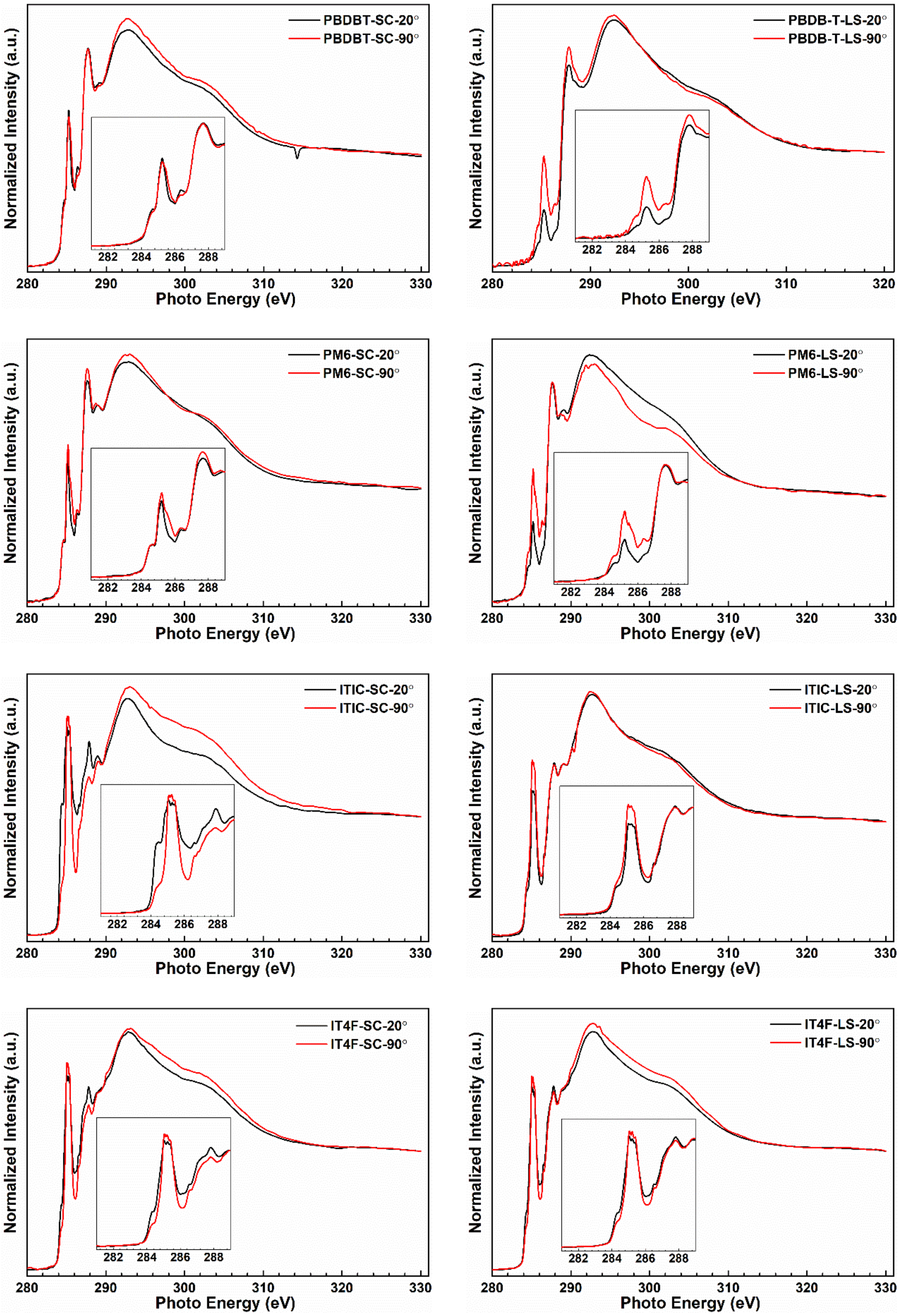

Supplementary Fig. 4 Angular dependence of C K-edge NEXAFS spectra in total electron yield (TEY) detection mode for four model material films (PBDB-T, PM6, ITIC, IT4F) made from spin-coating (SC) or Langmuir-Schäfer (LS) methods, with X-ray beam at 90° (normal incident), 20° (grazing incident) related to the sample surface.

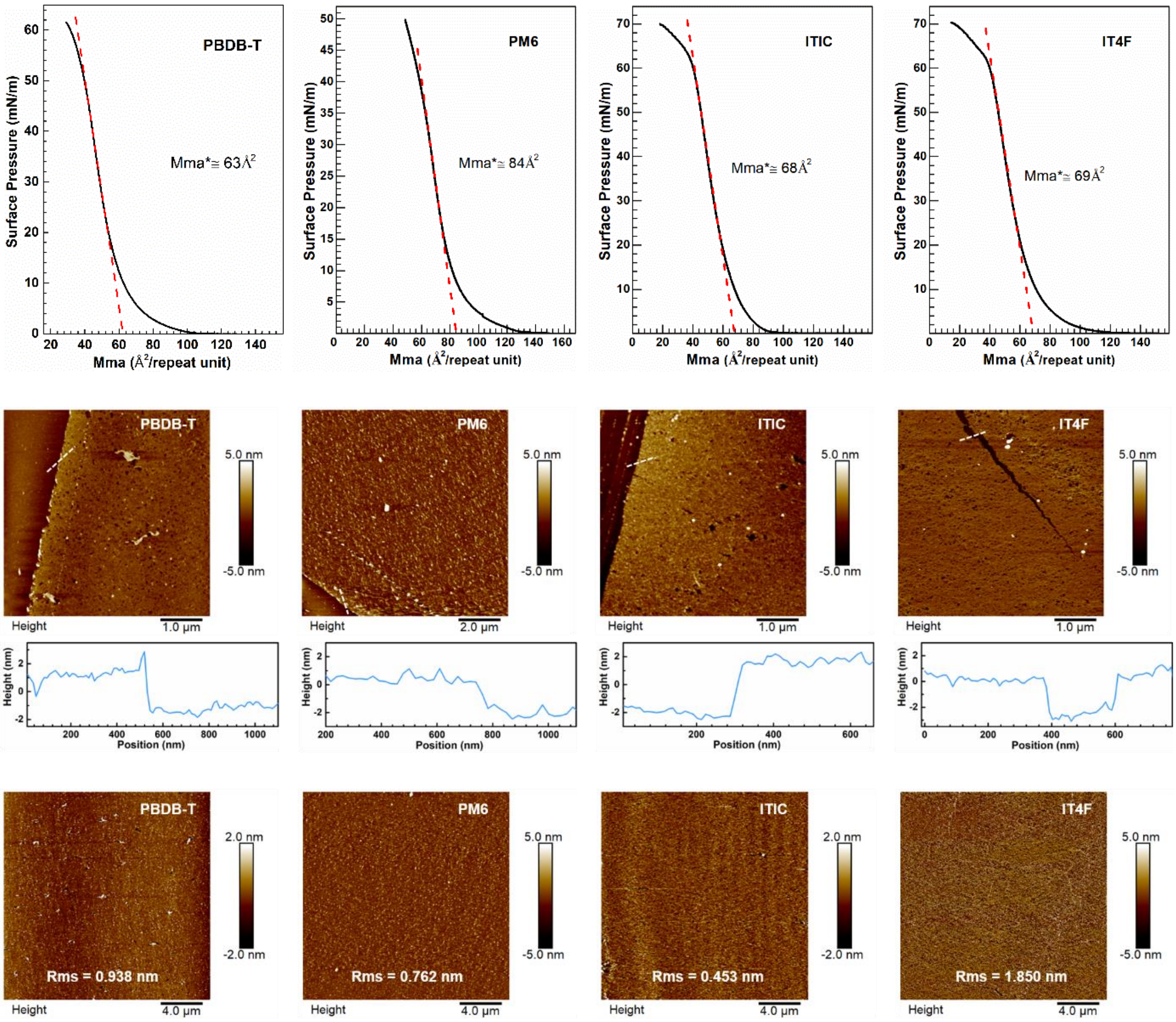

Supplementary Fig. 5 Top row: surface pressure versus mean monomeric area (Mma) isotherm (P-A-isotherm) of PBDB-T, PM6, ITIC, IT4F spread from solution on water surface, whose mean monomeric area (Mma*) determined from this isotherm is 63, 84, 68, 69 Å^2, respectively. Middle row: topography image of the LS monolayer film measured by AFM, and cross-section analysis along the scratch on film. The thickness (d) of the PBDB-T, PM6, ITIC, and IT4F monolayer is about 2.71, 2.87, 3.34 and 2.84 nm. Bottom row: root mean square roughness (Rms) of PBDB-T, PM6, ITIC, and IT4F monolayer film in 20 μm × 20 μm scale, and the roughness is 0.938, 0.762, 0.453 and 1.850 nm, respectively.

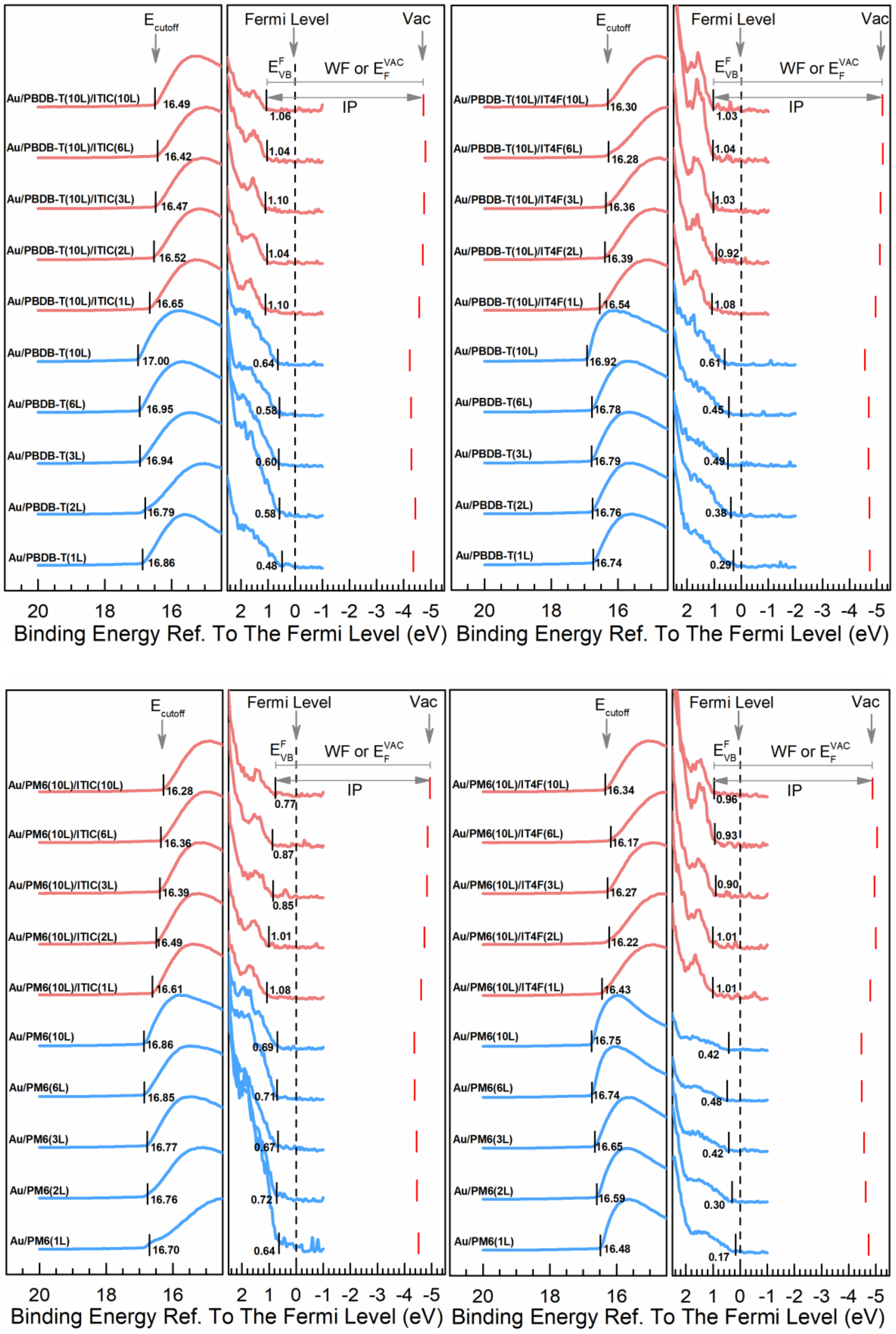

E_cutoff
Fermi Level
Vac
E_VB^F
WF or E_F^VAC
IP
Au/PBDB-T(10L)/ITIC(10L)
16.49
Au/PBDB-T(10L)/ITIC(6L)
16.42
Au/PBDB-T(10L)/ITIC(3L)
16.47
Au/PBDB-T(10L)/ITIC(2L)
16.52
Au/PBDB-T(10L)/ITIC(1L)
16.65
Au/PBDB-T(10L)
17.00
Au/PBDB-T(6L)
16.95
Au/PBDB-T(3L)
16.94
Au/PBDB-T(2L)
16.79
Au/PBDB-T(1L)
16.86
1.06
1.04
1.10
1.04
1.10
0.64
0.58
0.60
0.58
0.48
Au/PBDB-T(10L)/IT4F(10L)
16.30
Au/PBDB-T(10L)/IT4F(6L)
16.28
Au/PBDB-T(10L)/IT4F(3L)
16.36
Au/PBDB-T(10L)/IT4F(2L)
16.39
Au/PBDB-T(10L)/IT4F(1L)
16.54
Au/PBDB-T(10L)
16.92
Au/PBDB-T(6L)
16.78
Au/PBDB-T(3L)
16.79
Au/PBDB-T(2L)
16.76
Au/PBDB-T(1L)
16.74
1.03
1.04
1.03
0.92
1.08
0.61
0.45
0.49
0.38
0.29
20 18 16
2 1 0 -1 -2 -3 -4 -5
Binding Energy Ref. To The Fermi Level (eV)
Au/PM6(10L)/ITIC(10L)
16.28
Au/PM6(10L)/ITIC(6L)
16.36
Au/PM6(10L)/ITIC(3L)
16.39
Au/PM6(10L)/ITIC(2L)
16.49
Au/PM6(10L)/ITIC(1L)
16.61
Au/PM6(10L)
16.86
Au/PM6(6L)
16.85
Au/PM6(3L)
16.77
Au/PM6(2L)
16.76
Au/PM6(1L)
16.70
0.77
0.87
0.85
1.01
1.08
0.69
0.71
0.67
0.72
0.64
Au/PM6(10L)/IT4F(10L)
16.34
Au/PM6(10L)/IT4F(6L)
16.17
Au/PM6(10L)/IT4F(3L)
16.27
Au/PM6(10L)/IT4F(2L)
16.22
Au/PM6(10L)/IT4F(1L)
16.43
Au/PM6(10L)
16.75
Au/PM6(6L)
16.74
Au/PM6(3L)
16.65
Au/PM6(2L)
16.59
Au/PM6(1L)
16.48
0.96
0.93
0.90
1.01
1.01
0.42
0.48
0.42
0.30
0.17

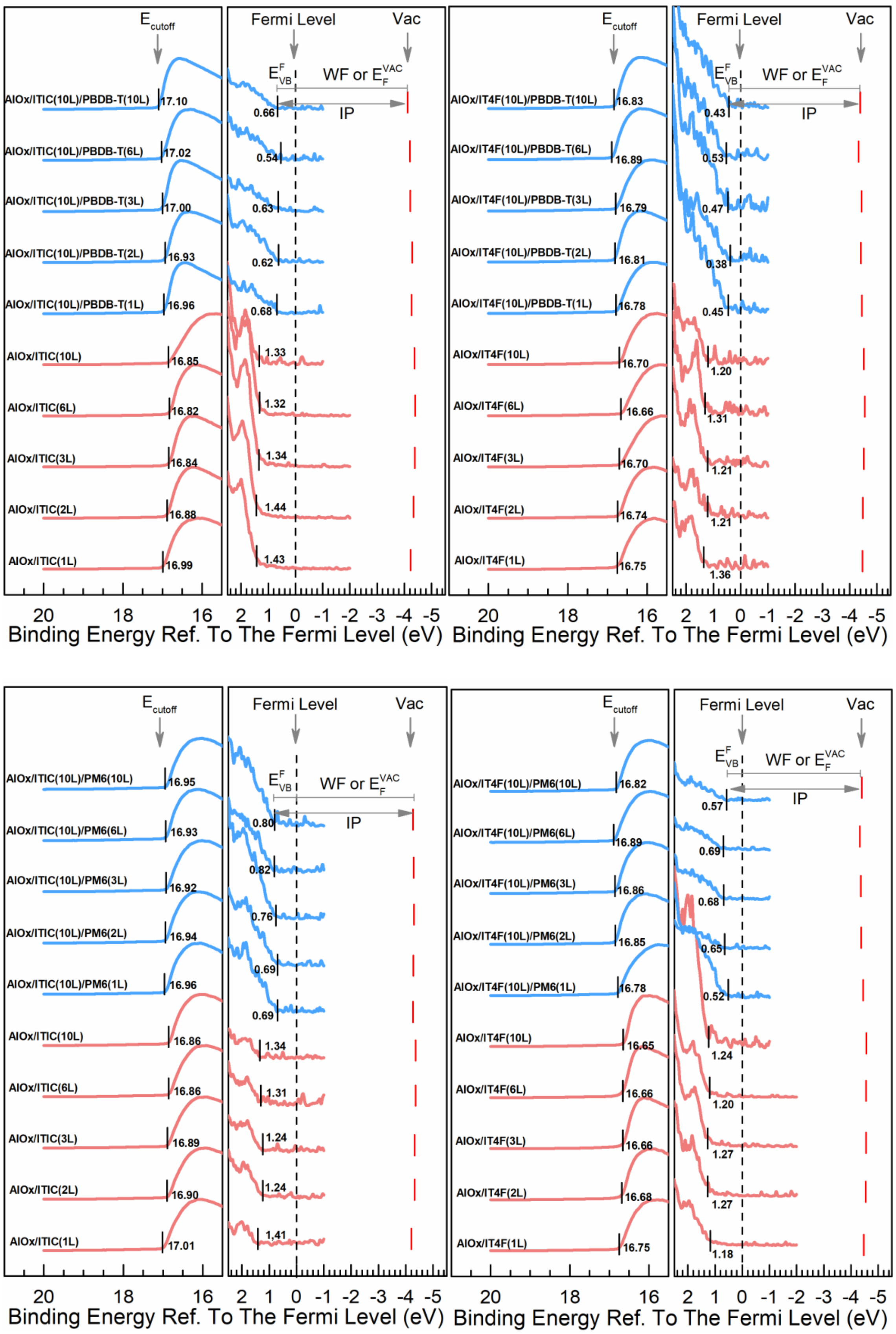

Supplementary Fig. 6 UPS spectra of D-A planar bilayer heterojunctions presented in a layer-by-layer manner. E_{cutoff} is the secondary-electron cutoff. E_{VB}^{F} is the energy of valence band (VB) edge referenced to the Fermi level. E_{F}^{Vac} is the energy of vacuum (Vac) level referenced to the Fermi level, which is equivalent to the work function (WF) of the sample.

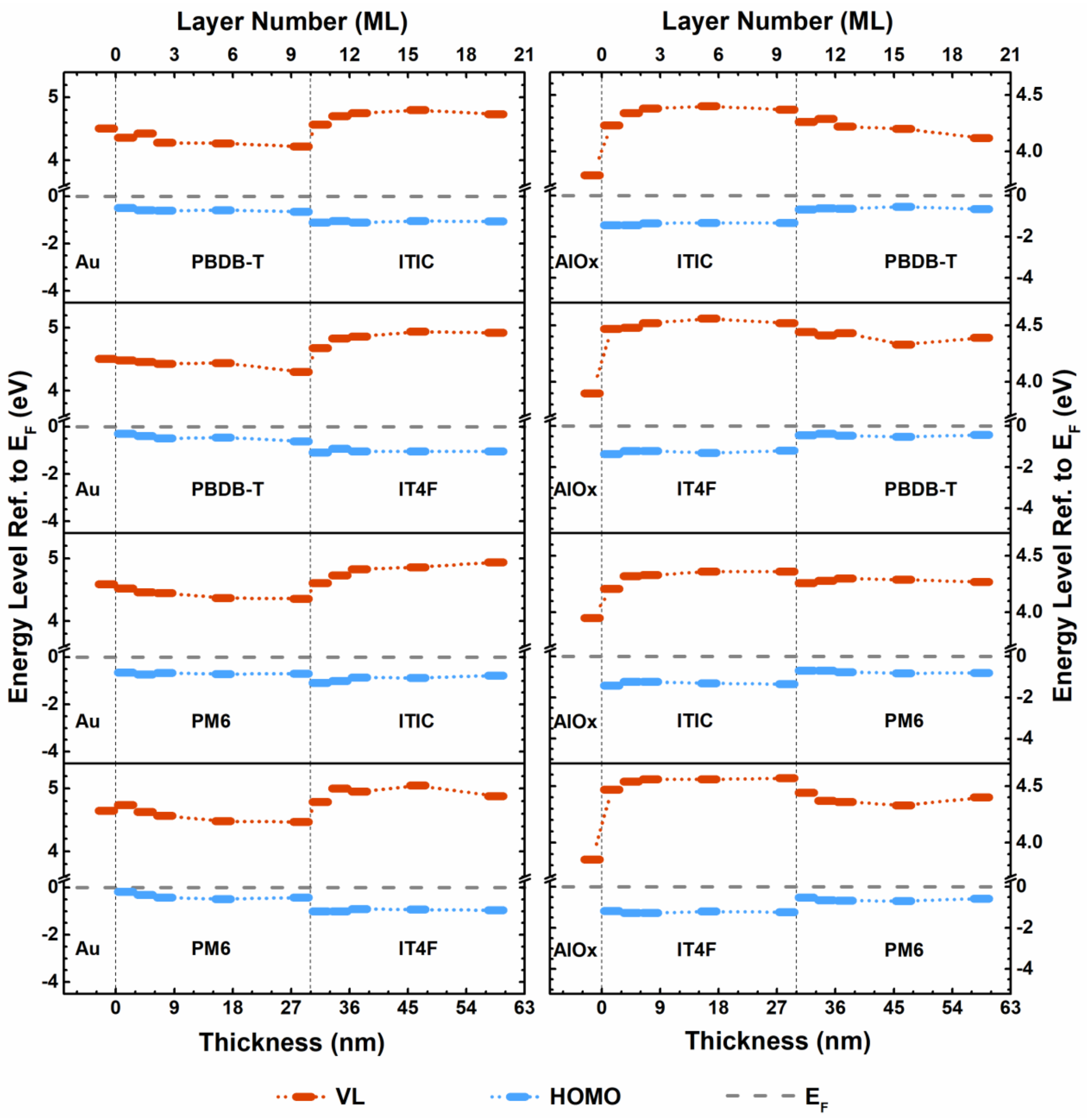

Supplementary Fig. 7 Energy level evolution during the monolayer-by-monolayer deposition process of planar D-A heterojunction. Vacuum level and HOMO level (both are referenced to E_F) of each monolayer are mapped versus the monolayer numbers (top axis) or the corresponding film thickness (bottom axis, presented by taking 3 nm as the average thickness of a monolayer). The left and right panel show the energy level evolutions in Au/D/A and AlO_x/A/D films, respectively.

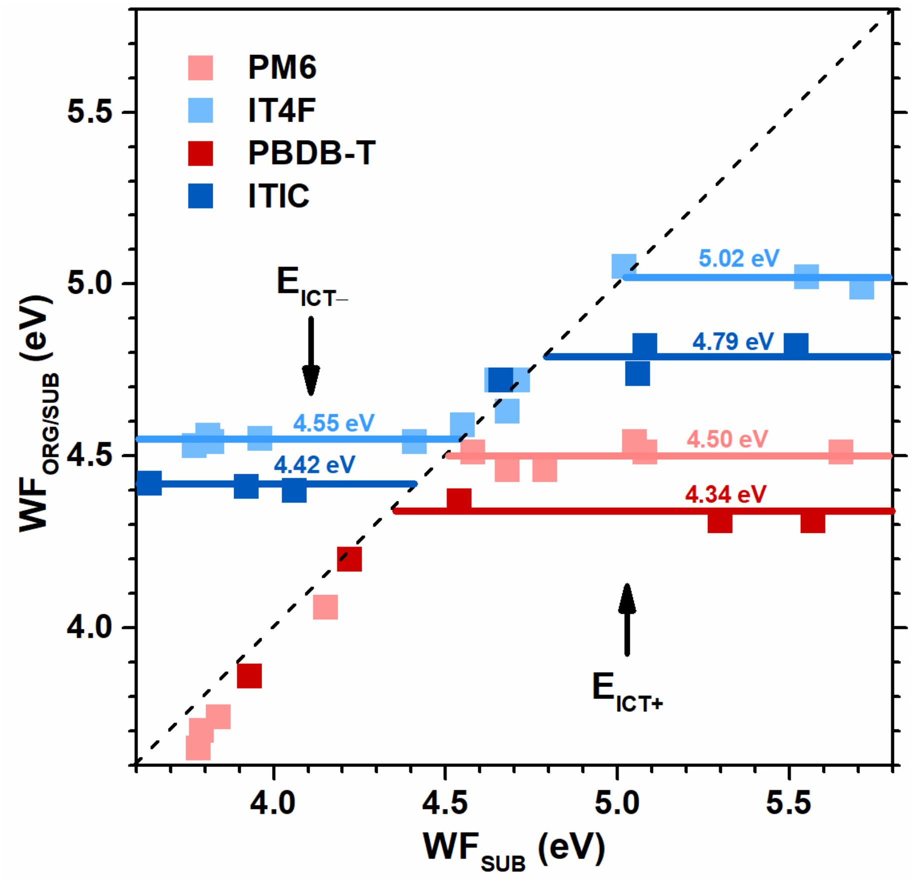

Supplementary Fig. 8 Pinning energies of donors (PM6, PBDB-T) and non-fullerene acceptors (ITIC, IT4F) determined by the dependences of the work function of films coated on substrates via solution process ($WF_{ORG/SUB}$) on the work function of bare substrate (WF_{SUB}). E_{ICT+}: positive pinning energy; E_{ICT-}: negative pinning energy.

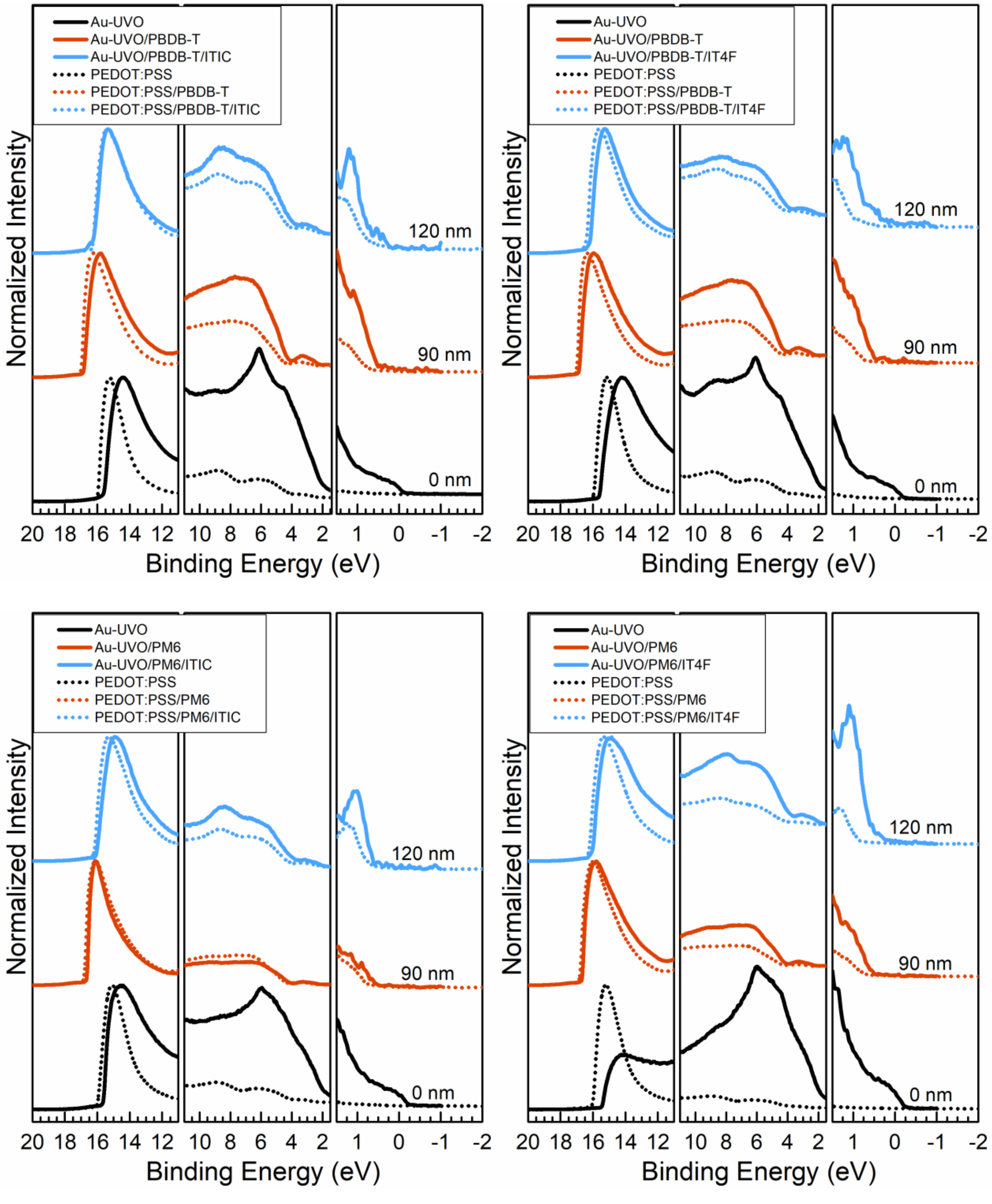

Au-UVO
Au-UVO/PBDB-T
Au-UVO/PBDB-T/ITIC
PEDOT:PSS
PEDOT:PSS/PBDB-T
PEDOT:PSS/PBDB-T/ITIC
Au-UVO/PBDB-T/IT4F
PEDOT:PSS/PBDB-T/IT4F
Au-UVO/PM6
Au-UVO/PM6/ITIC
PEDOT:PSS/PM6
PEDOT:PSS/PM6/ITIC
Au-UVO/PM6/IT4F
PEDOT:PSS/PM6/IT4F
Normalized Intensity
120 nm
90 nm
0 nm
Binding Energy (eV)

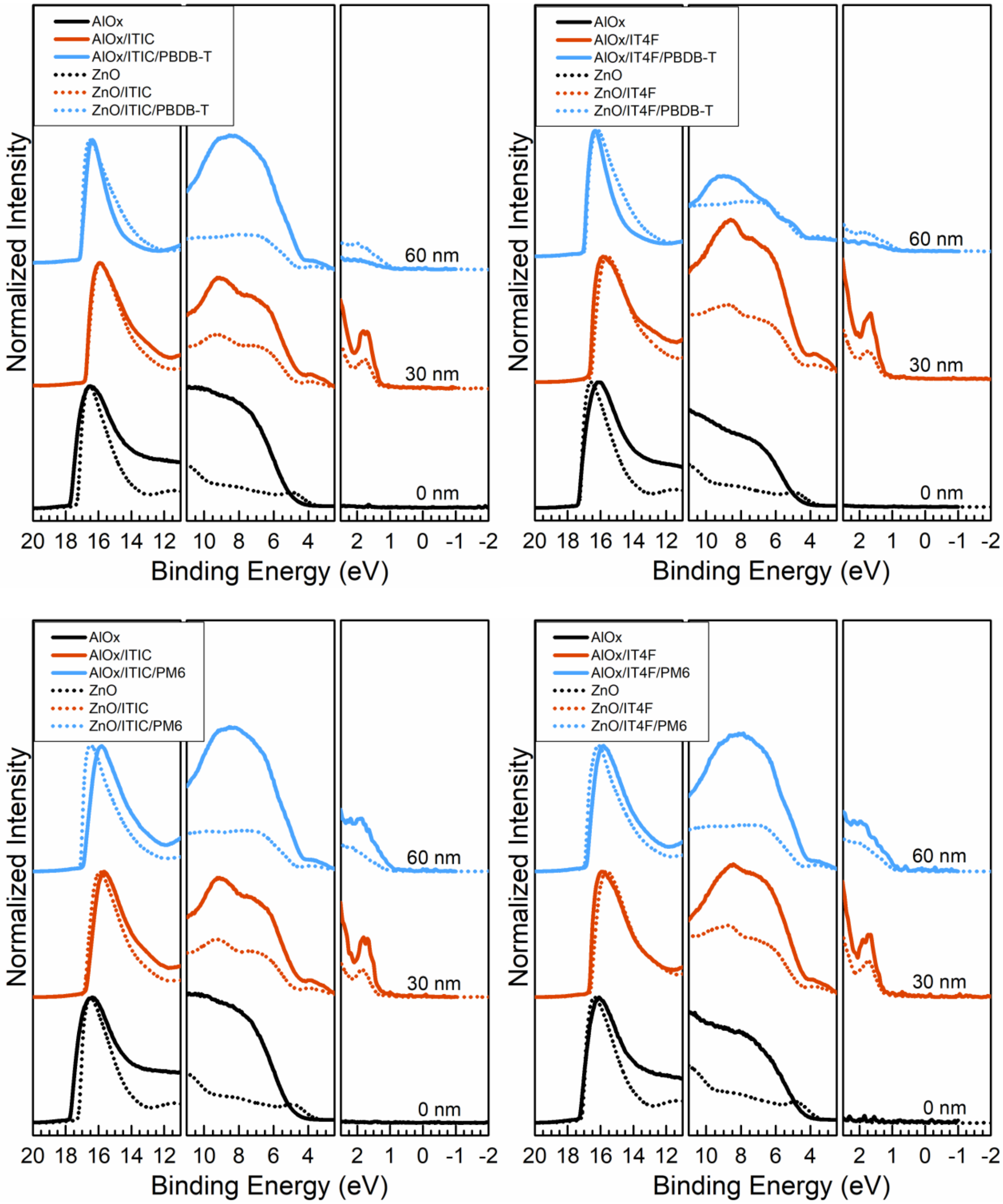

Supplementary Fig. 9 UPS spectra of substrates, substrate/D, substrate/A, substrate/D/A, substrate/A/D films. Substrate/D/A bilayer films are prepared by Spin-coating/Spin-coating (SC/SC) method, and Substrate/A/D films are prepared by Spin-coating/Surface-spreading (SC/SS) method. The error bar of the film thickness is ±10 nm.

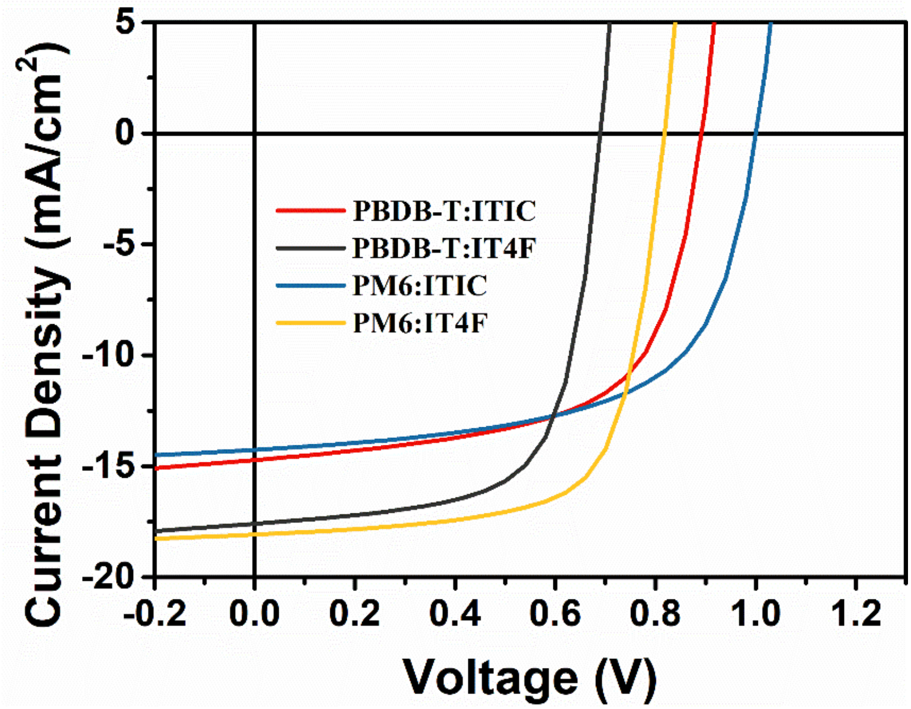

Supplementary Fig. 10 Current density−voltage characteristics of the devices based on PBDB-T:ITIC, PBDB-T:IT4F, PM6:ITIC, and PM6:IT4F in inverted structure under the illumination of a 470 nm LED.

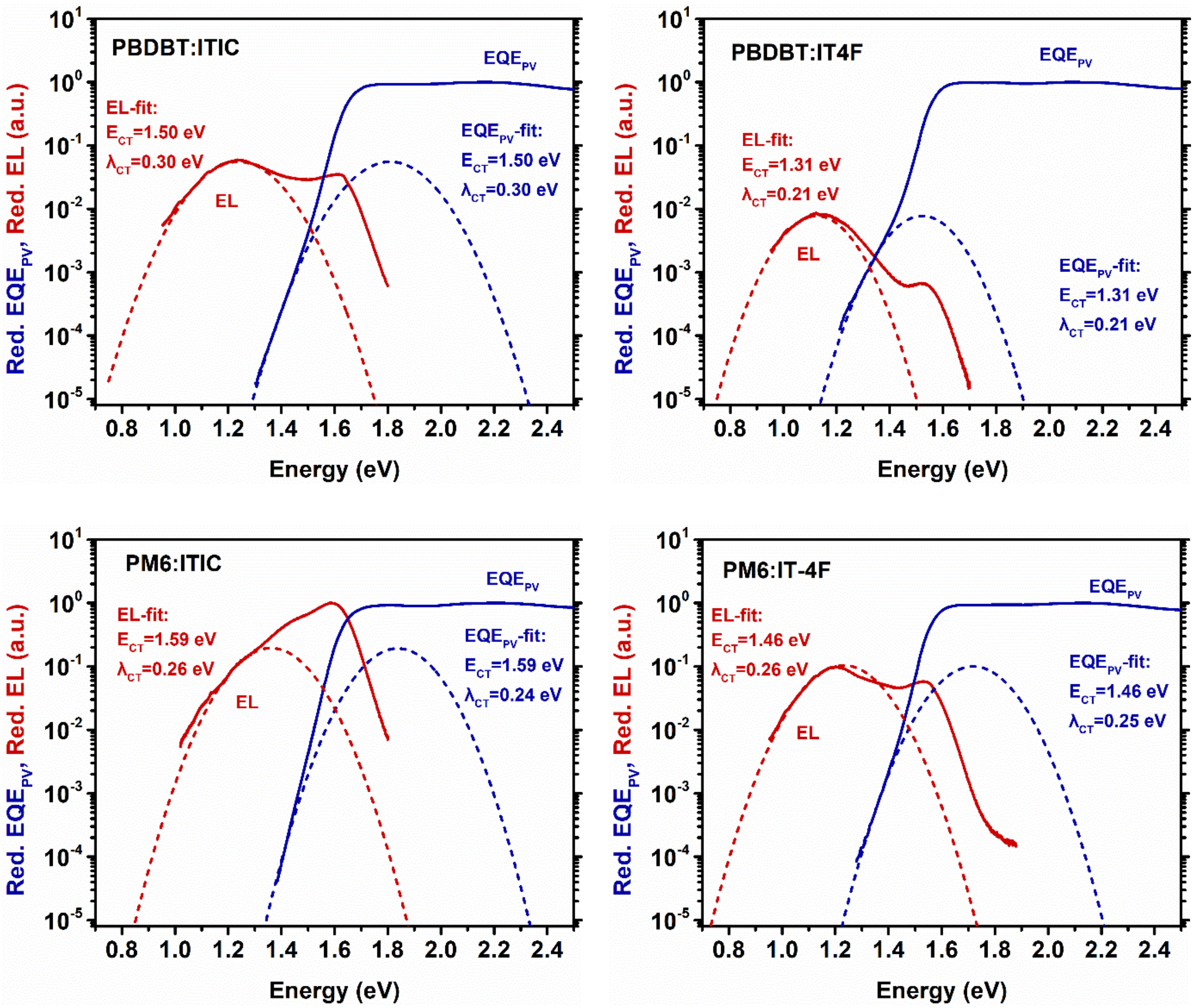

Supplementary Fig. 11 Normalized reduced EQE$_{PV}$ and reduced electroluminescence (EL) spectra (solid lines) of the four blend devices: PBDB-T:ITIC, PBDB-T:IT4F, PM6:ITIC, and PM6:IT4F. E_{CT} values are given by the crossing point between their corresponding fitting lines.

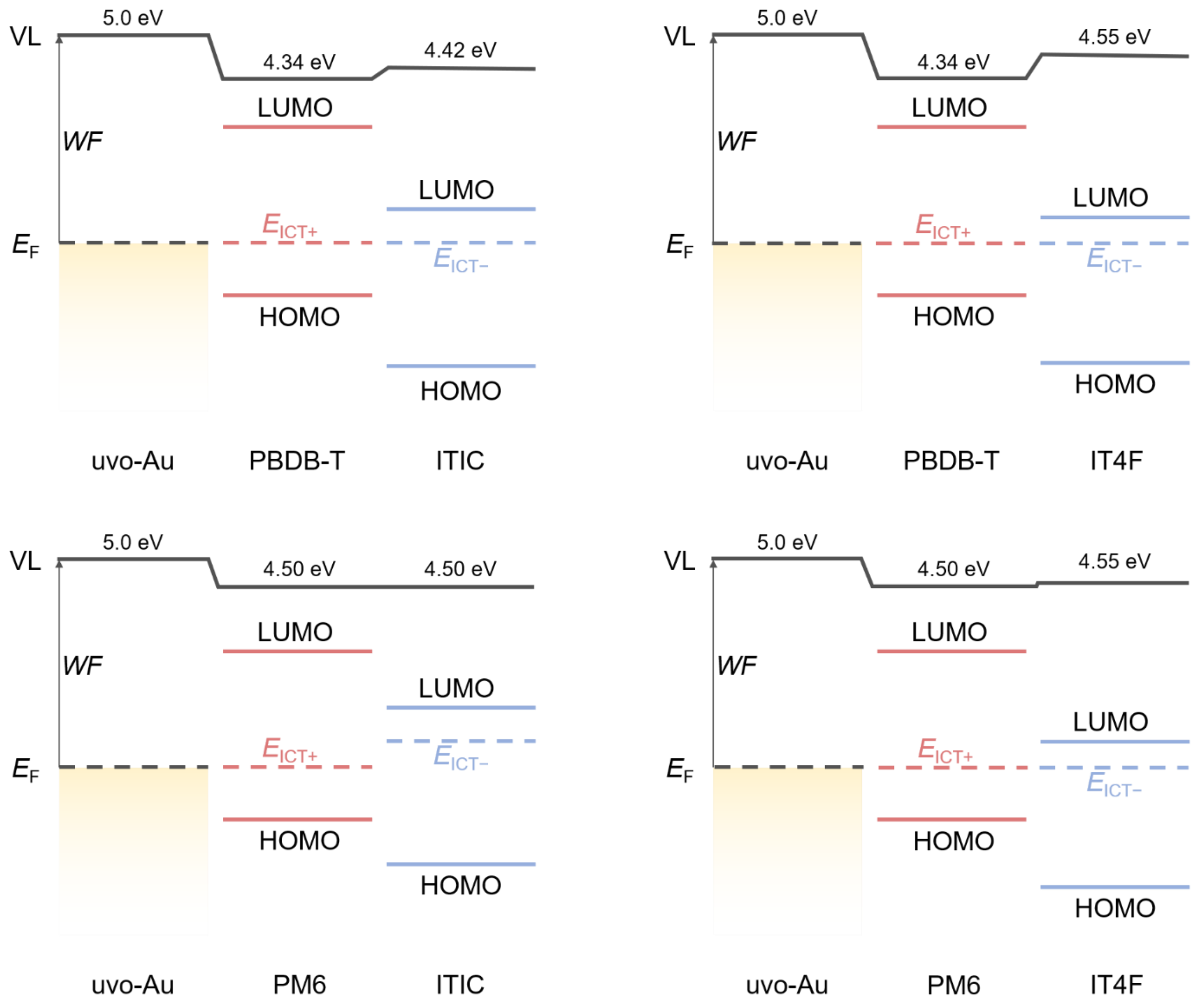

Supplementary Fig. 12 Estimated ELA for D-A interfaces of four model materials (ITIC, IT4F, PBDB-T, PM6) according to ICT model.

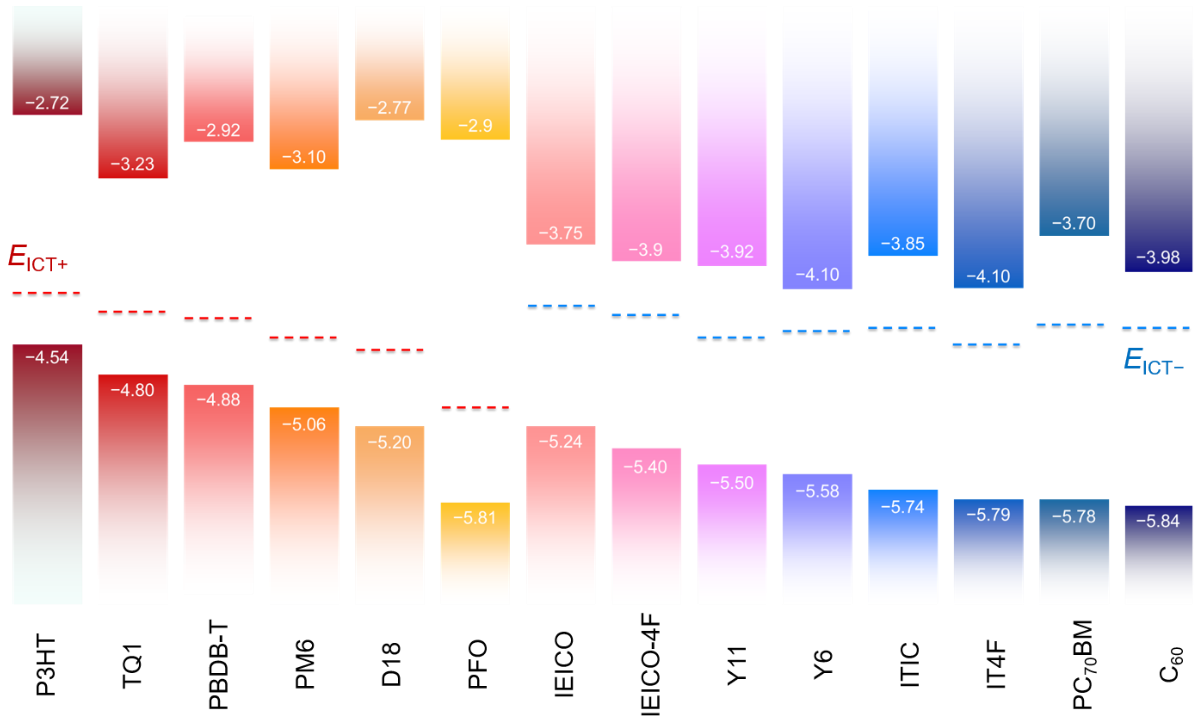

Supplementary Fig. 13 Energy levels measured from neat materials, where HOMOs are all measured by UPS in lab, and LUMOs are obtained from literatures either from IPES method (PM6, IEICO, IEICO-4F, Y6, ITIC, IT4F, $PC_{70}BM$, C_{60}),[4–6] or from CV method (P3HT, PBDB-T, D18, PFO, Y11). [7–11] LUMO of TQ1 is obtained by the combination of IP and the optical gap.[12]

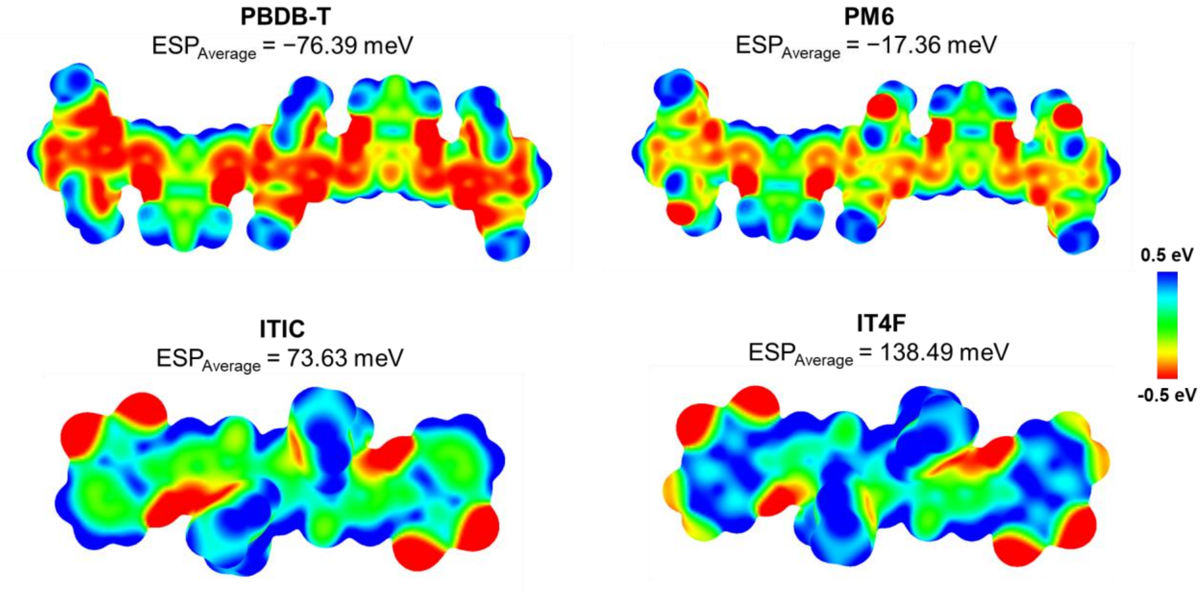

Supplementary Fig. 14 ESP distributions of the donor and acceptors (electron density isosurfaces of 0.001 au).

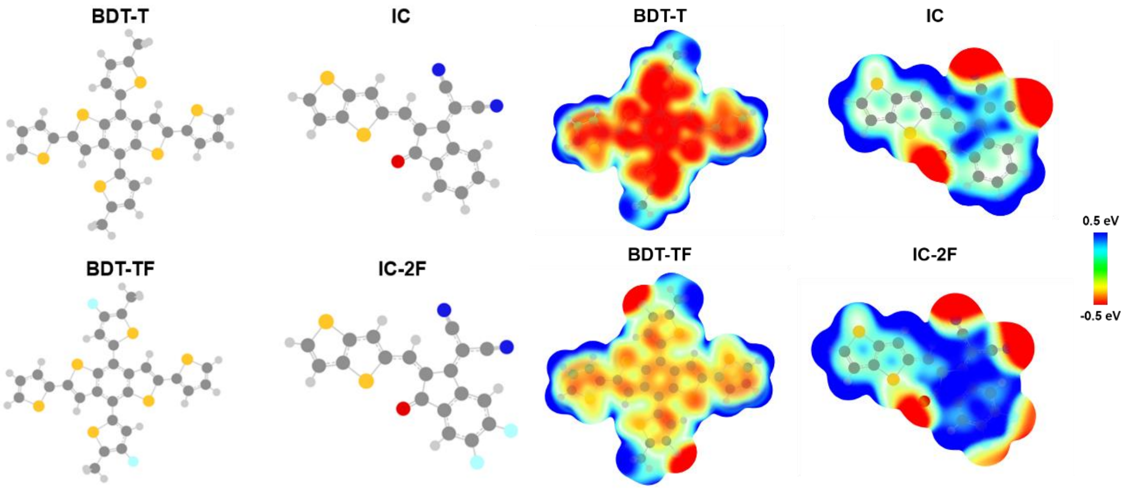

Supplementary Fig. 15 Rendering of the electrostatic potential on molecular surface for D units of PBDBT (BDT-T) and PM6 (BDT-TF), A units of ITIC (IC) and IT4F (IC-2F).

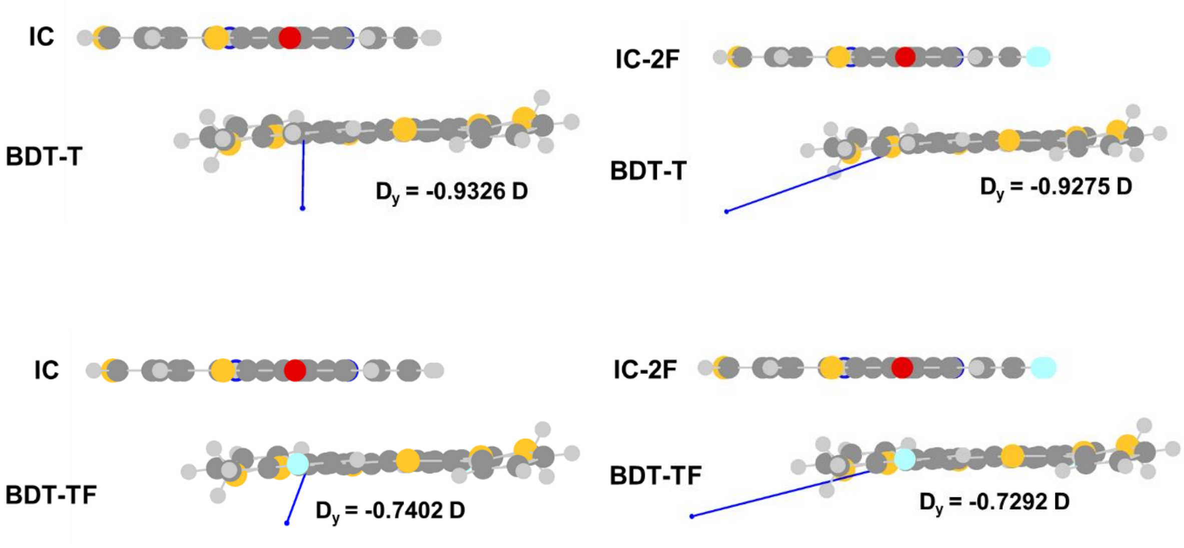

Supplementary Fig. 16 Electrostatic potential induced dipole calculation for D-A units with face-on/face-on orientation in the designated y direction (D_y). The π-π stacking space of 3.5 Å is adopted. The dipole unit is shown in Debye (D).

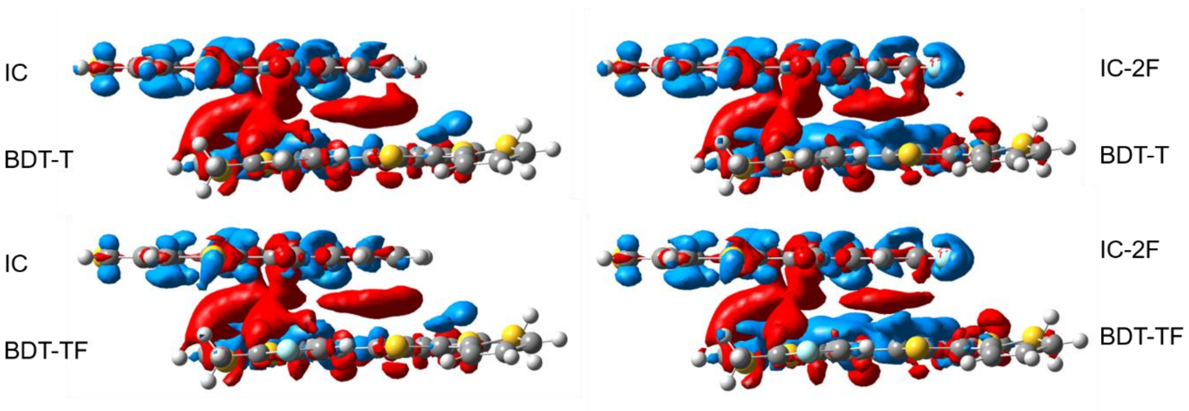

Supplementary Fig. 17 The electron/hole density distributions at interfaces of four D-A unit pairs at ground states. Hole and electron densities are presented in red and blue, receptively.

Supplementary Tables 1-8

Supplementary Table 1 Orientation determination of films from NEXAFS.

Films	Angular dependence of peak intensity		Orientation suggested from NEXAFS
	Shoulder peak (284.4 eV)	Main peak (285 eV)	
PBDB-T-SC	No angular dependence	No angular dependence	No preferential orientation or tilted roughly at magic angle (≈ 54.7°)
PBDB-T-LS	Intensity (90°) > Intensity (20°)	Intensity (90°) > Intensity (20°)	Preferential edge-on orientation
PM6-SC	No angular dependence	No angular dependence	No preferential orientation or tilted roughly at magic angle (≈ 54.7°)
PM6-LS	Intensity (90°) > Intensity (20°)	Intensity (90°) > Intensity (20°)	Preferential edge-on orientation
ITIC-SC	Intensity (20°) > Intensity (90°)	No angular dependence	Preferential face-on orientation (based on planar conformation assumption)
ITIC-LS	Intensity (90°) > Intensity (20°)	Intensity (90°) > Intensity (20°)	Preferential edge-on orientation
IT4F-SC	Intensity (20°) > Intensity (90°)	No angular dependence	Preferential face-on orientation but more disordered (based on planar conformation assumption)
IT4F-LS	No angular dependence	No angular dependence	No preferential orientation or tilted roughly at magic angle (≈ 54.7°)

Supplementary Table 2 Summary of the measured and calculated values for monolayer film of four model materials by LS method. l (molecular length) and w (molecular width) are measured from molecular coordinates in built molecule models. The calculated mean monomeric area ($Mma^*_{cal.}$) is the product of l (or w) with estimated π-π stacking distance. The measured $Mma^*_{mea.}$ is extrapolated at zero pressure by the red line of P-A-isotherm curve in Supplementary Fig. 5. $d_{cal.}$ (calculated monolayer film thickness) is estimated by adding 4 Å Van de Waals film-substrate distance to l (or w) of molecule or polymer segment. The measured film thickness ($d_{mea.}$) and root mean square roughness (Rms) are obtained from AFM.

Material	l (Å)	w (Å)	π-π stacking distance (Å)	$Mma^*_{cal.}$ (Å^2)	$Mma^*_{mea.}$ (Å^2)	$d_{cal.}$ (nm)	$d_{mea.}$ (nm)	Rms (nm)
PBDB-T	15.1	24.9	4	60.4 (99.6)	63	1.91 (2.89)	2.71	0.938
PM6	15.6	22.6	4	62.4 (90.4)	84	1.96 (2.66)	2.87	0.762
ITIC	28.7	23.8	3	86.1 (71.4)	68	3.27 (2.78)	3.34	0.453
IT4F	29.1	23.6	3	87.3 (70.8)	69	3.31 (2.76)	2.84	1.850

Supplementary Table 3 The average WF and IP values of neat multilayer films (Au/D, AlO_x/A) or D-A multilayer films (Au/D/A, AlO_x/A/D) calculated from the data points at equilibrium region of Fig. 2 in the main text, and WF shifts at D-A interface $\Delta WF_{D/A}$ ($\Delta WF_{D/A} = \overline{WF}_{Au/D/A} - \overline{WF}_{Au/D}$) or $\Delta WF_{A/D}$ ($\Delta WF_{A/D} = \overline{WF}_{AlOx/A/D} - \overline{WF}_{AlOx/A}$).

D/A Interfaces	$\overline{WF}_{Au/D}$	$\overline{WF}_{Au/D/A}$	$\overline{IP}_{Au/D}$	$\overline{IP}_{Au/D/A}$	$\Delta WF_{D/A}$
Au/PBDB-T/ITIC	4.30	4.75	4.89	5.78	+0.45
Au/PBDB-T/IT4F	4.41	4.89	4.87	5.87	+0.48
Au/PM6/ITIC	4.41	4.84	5.12	5.71	+0.43
Au/PM6/IT4F	4.54	4.97	4.94	5.90	+0.43
**A/D Interfaces**	$\overline{WF}_{AlOx/A}$	$\overline{WF}_{AlOx/A/D}$	$\overline{IP}_{AlOx/A}$	$\overline{IP}_{AlOx/A/D}$	$\Delta WF_{A/D}$
AlO_x/ITIC/PBDB-T	4.37	4.21	5.72	4.84	−0.16
AlO_x/IT4F/PBDB-T	4.52	4.39	5.77	4.85	−0.13
AlO_x/ITIC/PM6	4.34	4.29	5.62	5.03	−0.06
AlO_x/IT4F/PM6	4.56	4.37	5.77	5.00	−0.19

Supplementary Table 4 Comparison of the interface dipoles in D-A bilayer films on different substrates. D (bottom phase)/A (top phase) bilayer films are prepared by Spin-coating/Spin-coating (SC/SC) method, and A (bottom phase)/D (top phase) films are prepared by Spin-coating/Surface-spreading (SC/SS) method.

Substrate	Au	AlO_x	PEDOT:PSS	ZnO
D-A interfaces	$\Delta WF_{D/A}$	$\Delta WF_{A/D}$	$\Delta WF_{D/A}$	$\Delta WF_{A/D}$
PBDB-T:ITIC	+0.60	−0.33	+0.71	−0.27
PBDB-T:IT4F	+0.65	−0.36	+0.57	−0.37
PM6:ITIC	+0.60	−0.16	+0.62	−0.24
PM6:IT4F	+0.71	−0.20	+0.54	−0.33

Supplementary Table 5 Summary of detailed photovoltaic parameters of the devices. Values are averaged over ten devices.

Blend	E_g^{PV} (eV)	J_{SC} (mA cm^{-2})	FF (%)	V_{OC} (V)	PCE (%)
PBDB-T:ITIC	1.65	14.717	0.624	0.891	8.292
PM6:ITIC	1.64	14.262	0.616	1.000	8.787
PBDB-T:IT4F	1.56	17.587	0.666	0.690	8.077
PM6:IT4F	1.56	18.071	0.693	0.818	10.246

Supplementary Table 6 Summary of the pinning energies of materials.

Materials	E_{ICT+} (eV)	E_{ICT-} (eV)
P3HT (SC)	4.14	-
TQ1 (SC)	4.29	-
PBDB-T (SC)	4.34	-
PM6 (SC)	4.50	-
D18 (SC)	4.60	-
PFO (SC)	5.07	-
IEICO (SC)	-	4.24
IEICO-4F (SC)	-	4.33
Y11 (SC)	-	4.50
Y6 (SC)	-	4.46
ITIC (SC)	4.79	4.42
IT4F (SC)	5.02	4.55
$PC_{70}BM$ (SC)	-	4.40
C_{60}(evaporation)	-	4.42

Supplementary Table 7 Interface dipoles measured in other organic-organic heterojunctions.

Fabrication Method	SC/SC	SC/SS	LS/LS	LS/LS	SC/LS	SC/LS
D-A Interfaces	$\Delta WF_{D/A}$	$\Delta WF_{A/D}$	$\Delta WF_{D/A}$	$\Delta WF_{A/D}$	$\Delta WF_{D/A}$	$\Delta WF_{A/D}$
PBDB-T:Y6	+0.36 (crosslinked-SC/SC)	−0.41	+0.10	−0.20	+0.33	−0.05
PBDB-T:Y11	+0.39 (crosslinked-SC/SC)	−0.34	+0.43	−0.45	+0.66	−0.15
PM6:Y6	+0.16 (crosslinked-SC/SC)	−0.46	+0.27	−0.26	+0.35	−0.17
PM6:Y11	+0.24 (crosslinked-SC/SC)	−0.29	+0.49	−0.33	+0.45	−0.25
PBDB-T:IEICO	+0.33	-	-	-	-	-
PBDB-T:IEICO-4F	+0.39	-	-	-	-	-
PM6:IEICO	+0.10	-	-	-	-	-
PM6:IEICO-4F	+0.21	-	-	-	-	-
D18:IEICO	+0.06	-	-	-	-	-
D18:IT4F	+0.56	-	-	-	-	-
P3HT:ITIC	+0.58	-	-	-	+0.35	-
P3HT:IT4F	+0.48	-	-	-	-	-
TQ1:ITIC	+0.71	-	-	-	-	-
TQ1:IT4F	+0.70	-	-	-	-	-
PFO:ITIC	−0.01	-	-	-	+0.05	-
PFO:$PC_{70}BM$	+0.03	-	-	-	-	-
PBDB-T:$PC_{70}BM$	+0.26	-	-	-	-	-
PM6:$PC_{70}BM$	+0.20	-	-	-	-	-
PBDB-T:C_{60}	+0.10 (SC/evaporation)	-	-	-	-	−0.03 (evaporation/ LS)
PM6:C_{60}	+0.01 (SC/evaporation)	-	-	-	-	-
P3HT:C_{60}	0.28 (SC/evaporation)	-	-	-	-	-

Supplementary Table 8 Estimated D-A interface dipole matrix according to ICT model.

Acceptor \ Donor	P3HT	TQ1	PBDB-T	PM6	D18	PFO
IEICO	0.1	0	0	0	0	0
IEICO-4F	0.19	0.04	0	0	0	0
Y11	0.36	0.21	0.16	0	0	0
Y6	0.32	0.17	0.12	0	0	0
ITIC	0.28	0.13	0.08	0	0	0
IT4F	0.41	0.26	0.21	0.05	0	0
$PC_{70}BM$	0.26	0.11	0.06	0	0	0
C_{60}	0.28	0.13	0.08	0	0	0

Supplementary Note 1

In the C K-edge NEXAFS spectra of spin-coated (SC) ITIC or IT4F films (supplementary Fig.4), the frontier structures from 284–286 eV are corresponded to the C 1s → C=C π* transitions, and the shoulder structures at the lowest photo energy around 284.4 eV are corresponded to the frontier edge of the LUMO structures of acceptors. Moreover, the frontier LUMO density of states is believed to be dominated by the end groups of the acceptors (A part in the A-D-A structure), as the DFT calculation indicated in several literatures.[1,2] Thus, the orientation of the end groups in acceptors could be obtained from the spectral weight change of the shoulder peak at 284.4 eV with changing the incident angle, though the main C=C π* transition peak at 285 eV do not give clear information.

From the NEXAFS spectra of ITIC-SC film, the end groups of ITIC show obvious face-on orientation where the shoulder peak at 284.4 eV shows higher intensity at grazing incident angle (20°), and lower intensity at normal incidence (90°). Furthermore, a near-planar configuration between the end groups and core structure is the most probable conformation owing to the alternating single-double bonds, so we think the average orientation of ITIC-SC film could be inferred from the obvious angular dependance of the end groups. However, we do not believe that the ITIC molecules present absolute planar configuration without any end-group rotations, thus we describe the orientation of ITIC-SC film carefully as 'preferentially face-on oriented', and the face-on orientation of ITIC films are also observed by another group.[3] Similar but a little weaker angular dependence is observed on the NEXAFS spectra of IT4F-SC films. Thus IT4F-SC films are also preferentially face-on oriented but more disordered.

In contrast, NEXAFS spectra of the LS films all show obvious edge-on orientation where the frontier structures related to π* states show lower intensity at grazing incident angle (20°), and higher intensity at normal incidence (90°), except for that of the IT4F-LS film. No obvious change of the spectral weight related to π* states for the IT4F-LS film upon angle variation, which can be assigned to either a fully amorphous LS film or to molecular chains that are extended along the substrate surface tilted edge-on at roughly the magic angle (≈ 54.7°). Based on the properties of LS technique and AFM results, the formation of an amorphous IT4F film can be ruled out. Thus, the slightly less ordered tilted edge-on orientation is the most probable for the IT4F-LS film.

The summary for the orientation determination from NEXAFS spectra can be seen in the Supplementary Table 1.

Supplementary References

1. Gao, B. *et al.* Multi-component non-fullerene acceptors with tunable bandgap structures for efficient organic solar cells. *J. Mater. Chem. A* **6**, 23644–23649 (2018).
2. Mehboob, M. Y. *et al.* Designing of benzodithiophene core-based small molecular acceptors for efficient non-fullerene organic solar cells. *Spectrochim. Acta - Part A Mol. Biomol. Spectrosc.* **244**, 118873 (2021).
3. Du, Y. Y. *et al.* Electronic states and molecular orientation of ITIC film. *Chinese Phys. B* **27**, (2018).
4. Karuthedath, S. *et al.* Intrinsic efficiency limits in low-bandgap non-fullerene acceptor organic solar cells. *Nat. Mater.* **20**, 378–384 (2021).
5. Ratcliff, E. L. *et al.* Energy level alignment in PCDTBT:PC70BM solar cells: Solution processed NiOx for improved hole collection and efficiency. *Org. Electron.* **13**, 744–749 (2012).
6. Yoshida, H. Low-energy inverse photoemission study on the electron affinities of fullerene derivatives for organic photovoltaic cells. *J. Phys. Chem. C* **118**, 24377–24382 (2014).
7. Kadem, B., Fakher Alfahed, R. K., Al-Asadi, A. S. & Badran, H. A. Morphological, structural, optical, and photovoltaic cell of copolymer P3HT: ICBA and P3HT:PCBM. *Optik (Stuttg).* **204**, 164153 (2020).
8. Zhao, W. *et al.* Fullerene-free polymer solar cells with over 11% efficiency and excellent thermal stability. *Adv. Mater.* **28**, 4734–4739 (2016).
9. Liu, Q. *et al.* 18% Efficiency organic solar cells. *Sci. Bull.* **65**, 272–275 (2020).
10. Cleave, V. *et al.* Transfer processes in semiconducting polymer-porphyrin blends. *Adv. Mater.* **13**, 44–47 (2001).
11. Yuan, J. *et al.* Understanding energetic disorder in electron-deficient-core-based non-fullerene solar cells. *Sci. China Chem.* **63**, 1159–1168 (2020).
12. Bao, Q. *et al.* Intermixing effect on electronic structures of TQ1:PC71BM bulk heterojunction in organic photovoltaics. *Sol. RRL* **1**, 1700142 (2017).
