# Supplementary material for: Mapping the energy level alignment at donor/acceptor interfaces in non-fullerene organic solar cells
Source: Nat Commun. 2022 Apr 19;13:2046. doi: 10.1038/s41467-022-29702-w (PMC9018783; doi:10.1038/s41467-022-29702-w)
Supplement: Supplementary file 3 — Solar Cells Reporting Summary [file 41467_2022_29702_MOESM3_ESM.pdf]

nature research

Corresponding author(s): Xian'e Li; Mats Fahlman

Solar Cells Reporting Summary

Nature Research wishes to improve the reproducibility of the work that we publish. This form is intended for publication with all accepted papers reporting the characterization of photovoltaic devices and provides structure for consistency and transparency in reporting. Some list items might not apply to an individual manuscript, but all fields must be completed for clarity.

For further information on Nature Research policies, including our data availability policy, see Authors & Referees.

~ Experimental design

Please check: are the following details reported in the manuscript?

1. Dimensions

Item	Yes	No	Response
Area of the tested solar cells	x		Described in Methods, section "Device fabrication".
Method used to determine the device area	x		Described in Methods, section "Device fabrication".

2. Current-voltage characterization

Item	Yes	No	Response
Current density-voltage (J-V) plots in both forward and backward direction		x	Just J-V plot in forward direction since there is no hysteresis in organic solar cell
Voltage scan conditions<br>*For instance: scan direction, speed, dwell times*	x		Section "Current density–voltage characteristics (J–V)".
Test environment<br>*For instance: characterization temperature, in air or in glove box*	x		Section "Current density–voltage characteristics (J–V)".
Protocol for preconditioning of the device before its characterization	x		Section "Current density–voltage characteristics (J–V)".
Stability of the J-V characteristic<br>*Verified with time evolution of the maximum power point or with the photocurrent at maximum power point; see ref. 7 for details.*		x	Not verified, because the stability of solar cells is not a critical issue for the claims and conclusions of this study.

3. Hysteresis or any other unusual behaviour

Item	Yes	No	Response
Description of the unusual behaviour observed during the characterization		x	No unusual behavior has been observed.
Related experimental data		x	No unusual behavior has been observed.

4. Efficiency

Item	Yes	No	Response
External quantum efficiency (EQE) or incident photons to current efficiency (IPCE)	x		Provided in Supplementary Information Figure 7.
A comparison between the integrated response under the standard reference spectrum and the response measure under the simulator		x	Not relevant to this study
For tandem solar cells, the bias illumination and bias voltage used for each subcell		x	Tandem solar cells are not reported in this study.

5. Calibration

Item	Yes	No	Response
Light source and reference cell or sensor used for the characterization	x		Section "Current density–voltage characteristics (J–V)".
Confirmation that the reference cell was calibrated and certified	x		Section "Current density–voltage characteristics (J–V)".

November 2017

Calculation of spectral mismatch between the reference cell and the devices under test	☐ Yes ☒ No	Not relevant to this study.

6. Mask/aperture

Size of the mask/aperture used during testing	☐ Yes ☒ No	Device area has been clarified in Methods, section "Device fabrication".
Variation of the measured short-circuit current density with the mask/aperture area	☐ Yes ☒ No	The influence of device area is not related to this study, but we have kept the device area always being the same.

7. Performance certification

Identity of the independent certification laboratory that confirmed the photovoltaic performance	☐ Yes ☒ No	Not applicable.
A copy of any certificate(s)<br>*Provide in Supplementary Information*	☐ Yes ☒ No	Not applicable.

8. Statistics

Number of solar cells tested	☒ Yes ☐ No	Number of cells tested is provided in Supplementary Table 3.
Statistical analysis of the device performance	☒ Yes ☐ No	Statistical results of the devices are listed in Supplementary Table 3.

9. Long-term stability analysis

Type of analysis, bias conditions and environmental conditions<br>*For instance: illumination type, temperature, atmosphere humidity, encapsulation method, preconditioning temperature*	☐ Yes ☒ No	Not analysed, because long-term stability is not a critical issue for the claims and conclusions of this study.
